# RNA-Binding Proteins CLK1 and POP7 as Biomarkers for Diagnosis and Prognosis of Esophageal Squamous Cell Carcinoma

**DOI:** 10.3389/fcell.2021.715027

**Published:** 2021-09-09

**Authors:** Xiuping Yang, Baoai Han, Zuhong He, Ya Zhang, Kun Lin, Hongguo Su, Davood K. Hosseini, Haiying Sun, Minlan Yang, Xiong Chen

**Affiliations:** ^1^Department of Otorhinolaryngology, Head and Neck Surgery, Zhongnan Hospital of Wuhan University, Wuhan, China; ^2^Department of Otorhinolaryngology, Union Hospital, Tongji Medical College, Huazhong University of Science and Technology, Wuhan, China; ^3^Department of Internal Medicine, Hackensack University Medical Center, Hackensack, NJ, United States

**Keywords:** ESCC, RBPs, OS, The Cancer Genome Atlas, CLK1, POP7

## Abstract

The abnormality of RNA-binding proteins (RBPs) is closely related to the tumorigenesis and development of esophageal squamous cell carcinoma (ESCC), and has been an area of interest for research recently. In this study, 162 tumors and 11 normal samples are obtained from The Cancer Genome Atlas database, among which 218 differentially expressed RBPs are screened. Finally, a prognostic model including seven RBPs (CLK1, DDX39A, EEF2, ELAC1, NKRF, POP7, and SMN1) is established. Further analysis reveals that the overall survival (OS) rate of the high-risk group is lower than that of the low-risk group. The area under the receiver operating characteristic (ROC) curve (AUC) of the training group and testing group is significant (AUCs of 3 years are 0.815 and 0.694, respectively, AUCs of 5 years are 0.737 and 0.725, respectively). In addition, a comprehensive analysis of seven identified RBPs shows that most RBPs are related to OS in patients with ESCC, among which EEF2 and ELCA1 are differentially expressed at the protein level of ESCC and control tissues. CLK1 and POP7 expressions in esophageal cancer tumor samples are undertaken using the tissue microarray, and show that CLK1 mRNA levels are relatively lower, and POP7 mRNA levels are higher compared with non-cancerous esophageal tissues. Survival analysis reveals that a higher expression of CLK1 predicts a significant worse prognosis, and a lower expression of POP7 predicts a worse prognosis in esophageal cancer. These results suggest that CLK1 may promote tumor progression, and POP7 may hinder the development of esophageal cancer. In addition, gene set enrichment analysis reveals that abnormal biological processes related to ribosomes and abnormalities in classic tumor signaling pathways such as TGF-β are important driving forces for the occurrence and development of ESCC. Our results provide new insights into the pathogenesis of ESCC, and seven RBPs have potential application value in the clinical prognosis prediction of ESCC.

## Introduction

Esophageal cancer is the eighth common cancer in the world and the sixth leading cause of cancer-related deaths (Ferlay et al., 2019; Macedo-Silva et al., 2019; Lin et al., 2020; Zeng et al., 2021). Esophageal cancer is mainly divided into two subtypes, esophageal squamous cell carcinoma (ESCC) and esophageal adenocarcinoma (EAC). The former one accounts for nearly 90% of cases and is the main subtype in Asia and East Africa. However, the recent epidemiology shows that the incidence rate of EAC has increased by 3–4 times, and its proportion is increasing among patients with esophageal cancer (Harada et al., 2020; Mikuni et al., 2021; Thrift, 2021). In western countries, EAC incidence has exceeded ESCC and has become the main histological type (Arnold et al., 2015; Thrift, 2021). At present, the molecular mechanism of the occurrence and development of ESCC has not been fully elucidated, and the prognosis of patients with ESCC is still poor, with a 5-years survival rate of less than 30% (Mariette et al., 2005). Therefore, new diagnostic methods for the early detection and early intervention of esophageal cancer must be explored.

RNA-binding protein (RBP) is a kind of protein that binds to RNA in RNA regulation. At present, more than 1500 RBPs are encoded in the human genome, accounting for 7.5% of protein coding genes (Uchida et al., 2019; Seo and Choi, 2021). As a trans acting factor, RBP and RNA recognition and interaction are mainly mediated by specific RNA binding domain (RBD) (Ostareck and Ostareck-Lederer, 2019). RBD is a structure with unique characteristics. At present, more than 600 types of RBD with different structures have been found in human beings, such as K-homology motif, RNA recognition motif, and zinc finger domain. In addition, an auxiliary domain on RBP mediates the protein–protein interaction (PPI). One or more RBD domains and one or more auxiliary domains are assembled together in different ways to form different RBPs (Hattori et al., 2016). RNA is combined with RBP to form ribonucleoprotein (RNP) complex to regulate gene expression. RBP has many effects on the binding target RNA, including splicing, modification, transportation, localization, stability, degradation, and translation (Attig and Ule, 2019). Therefore, the defect or obstruction of RNP formation affects the occurrence and development of the disease (Sephton and Yu, 2015). RBP also plays an important role in the occurrence and development of cancer (Pelava et al., 2016). Cancer is a complex, heterogeneous disease. Tumor cells can regulate the level of protein expression by hijacking post transcriptional regulation, such that they can better adapt to the microenvironment. Studies have found that RBP is dysregulated in different types of cancer, which affects the expression and function of oncoproteins and tumor suppressor proteins. For example, the deletion of insulin-like growth factor 2 mRNA binding protein 1 (IMP1) can promote the occurrence and development of colon tumor microenvironment (Mongroo et al., 2011; Hamilton et al., 2015). However, the increased copy number of negative elongation factor E in HCC can lead to the over activation of MYC signaling pathway and promote the progression of HCC (Dang et al., 2017). Therefore, deciphering the intricate interaction network between RBP and its cancer-related RNA targets will provide a better understanding of tumor biology and may reveal new cancer treatment targets.

At present, the research on RBPs in esophageal cancer is rare. Thus, this study attempts to analyze the potential value of RBPs in ESCC systematically by integrating a full set of RNA related proteins (RBPs) with clinical data obtained from The Cancer Genome Atlas (TCGA) portal. The expression profile analysis based on the gene chip provides convenience for the quantitative evaluation of esophageal cancer biology and measures the gene expression level in the whole genome. It paves the way for individualized therapy based on gene expression profiles, overcomes the heterogeneity of ESCC, and improves the therapeutic effect. First, the risk prediction model of the differentially expressed RBPs related genes (DERBPs) was constructed by identifying the DERBPs in ESCC. Then, Lasso regression and Cox regression analysis were used to optimize the model, and DERBPs related to overall survival (OS) were selected. These DERBPs were used to establish a Cox regression model (OS model), and ROC curve analysis was used to evaluate the specificity and sensitivity of the model. Human tissue microarray was used to verify the results, which showed that these specific models can accurately predict the prognosis of patients. These findings provide a new understanding of the pathogenesis of the disease and an effective multidimensional strategy based on biomarkers for the prognosis prediction of patients with ESCC.

## Materials and Methods

### RBP Expression Data Set and Preprocessing

The RNA sequencing data and corresponding clinical data of 162 ESCC and 11 normal esophageal tissue specimens were obtained from TCGA. A total of 1542 RBPs present in the human genome were extracted from the literature (Gerstberger et al., 2014). The expression log2 (x + 1) was used to standardize the RNA sequence map of ESCC. DESeq2 software package in R was used to evaluate the differential expression of RBP between ESCC and non-tumor specimens, and genes with at least 1.5-fold change and a corresponding *P*-value less than 0.05 were selected as DERBPs with significant differential expression. The heatmap package in R was used to cluster the DERBPs.

### Gene Ontology Function and Pathway Enrichment Analysis, PPI Network Construction, and Key Module Screening

The clusterProfiler package in R was used to perform gene ontology (GO) enrichment and Kyoto Encyclopedia of Genes and Genomes (KEGG) pathway analysis on DERBPs, and the main biological characteristics of these RBPs were comprehensively tested. The GO analysis term included three parts: biological process, molecular function, and cell composition. *P* < 0.05 and FDR < 0.05 were statistically significant. STRING database^1^ was used to identify the PPI information of the DERBPs. Then, the PPI network was further constructed and visualized by using Cytoscape 3.6.1 software. Finally, the molecular complex detection (MCODE) plugin was used to screen out important modules and genes in the PPI network, and the MCODE score and the number of nodes were greater than 5.

### Construction of the ESCC Prognostic Model

The significantly DERBPs data were integrated with the corresponding clinical information, the RBPs coexpressed in the PPI network were screened out, and then the data were randomly divided into training group and testing group for subsequent verification. The expression data of 197 RBPs in the training group were analyzed by univariate Cox regression, and the RBPs significantly correlated with survival rate were obtained (*P* < 0.05). Then, Lasso regression analysis was used to eliminate false positive parameters caused by overfitting. Finally, multivariate Cox regression analysis was used to determine seven prognostic RBPs and their coefficients, and then a prognostic model was constructed.

### Validation of the ESCC Prognostic Model

Risk score is an index to measure the prognosis risk of each patient with ESCC. The risk score of each patient in the training group and testing group was calculated according to the regression coefficient and expression value of each gene in the prognosis model. The calculation formula is as follows: β1^∗^Exp1 + β2^∗^Exp2 + βi^∗^Expi, where β represents the coefficient value, and Exp represents the gene expression level. Then, the patients with ESCC were divided into high-risk group and low-risk group based on the median risk score of the training group, the Kaplan–Meier (K–M) survival curve was drawn, and the difference of the OS rate between the two groups was evaluated by log rank test to determine statistical significance. In addition, a receiver operating characteristic (ROC) curve was generated to determine the accuracy of the prediction model. The area under the curve (AUC) value greater than or equal to 0.70 is the effective predictive value, and the AUC value greater than or equal to 0.6 is the acceptable predictive value. Finally, RMS R package was used for nomogram analysis to predict the possibility of OS. *P* < 0.05 was significant difference.

### Comprehensive Analysis of RBPs in the ESCC Prognostic Model

Correlation analysis was performed on the seven selected RBPs in the Tumor immune estimation resource (TIMER) online database, and the Pearson correlation coefficient between each gene pair was calculated. Then, the online database Gene expression profiling interactive analysis 2 (GEPIA2)^2^ was utilized to analyze the survival of RBPs in the risk-specific model, and the log rank test was used to evaluate the prognosis value. Logrank *P* ≤ 0.05 is a significant difference. Subsequently, by comparing the immunohistochemical staining images in the human protein Atlas database,^3^ the expression of the selected RBPs at the translation level was analyzed. Based on staining intensity, it was marked as high, medium, low, and undetected. cBioPortal online database was used to analyze the mutation and copy number changes of seven RBPs in the risk-specific model further.^4^ Finally, gene set enrichment analysis (GSEA) was performed to evaluate the potential mechanism of RBPs involved in esophageal carcinogenesis. The differences in pathways and corresponding biomarkers between high-risk and low-risk patients were determined. The normalized enrichment score (NES) and the normalized *P*-value were used to calculate the enrichment of the marker and the canonical path. Terms with |NES| > 1 and *P* < 0.05 were considered significantly rich.

### Microarray Analysis

Human esophageal cancer/adjacent tissue cDNA microarray is a cDNA array that detects the expression of target genes by the real-time fluorescence quantitative method. The cDNA was obtained from esophageal cancer tissues and corresponding adjacent tissues of patients with esophageal cancer. The expression of CLK1 and POP7 in esophageal carcinoma (*n* = 28) was analyzed, and adjacent normal tissues were matched (*n* = 28). Twenty-eight pairs of esophageal cancer cDNA were obtained from me-hesos095su01 tissue cDNA array (Shanghai, China). The mRNA levels of CLK1, POP7, and GAPDH were detected by ABI 7,500 rapid real-time PCR system (Applied Biosystems, Carlsbad, CA, United States) and SYBR Premix Ex Tag (Takara). Relative mRNA expression was determined using the cycle threshold (CT) formula 2^–Δ^ ^CT^, whereΔCT = [CT(target gene)—CT(GAPDH)]. The expression level was standardized with endogenous GAPDH. The specific primers were as follows: human CLK1, forward 5′-AGAGACCATGAAAGCCGGTAT-3′, reverse 5′-CATGTGAACGACGATGTGAAGT-3′; human POP7, forward 5′-GGTGCTGTGGAGGCTGAACT-3′, reverse 5′-GCCCAAGCCGTGAATGTAGAT-3′; Human GAPDH, forward 5′-TGC-ACC-AAC-TGC-TTA-GC-3′, and reverse 5′-AGC-TCA-GGGATG ACC TTG CC-3′.

## Results

### Differential Expression and Functional Annotation of RBPs in ESCC

**FIGURE 1:**
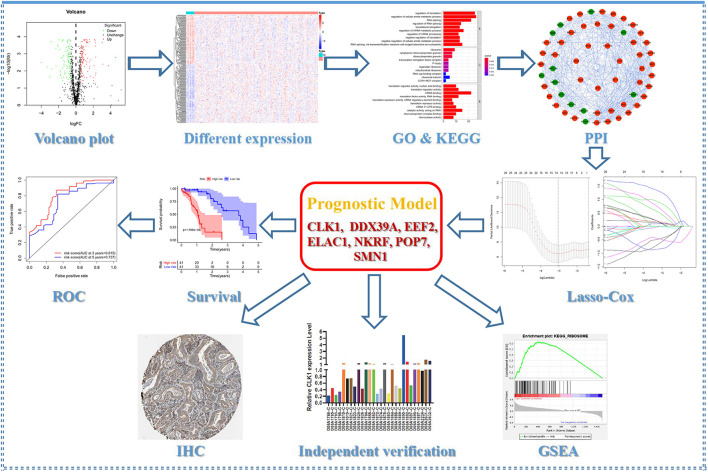
Flow chart of this study.

**FIGURE 2:**
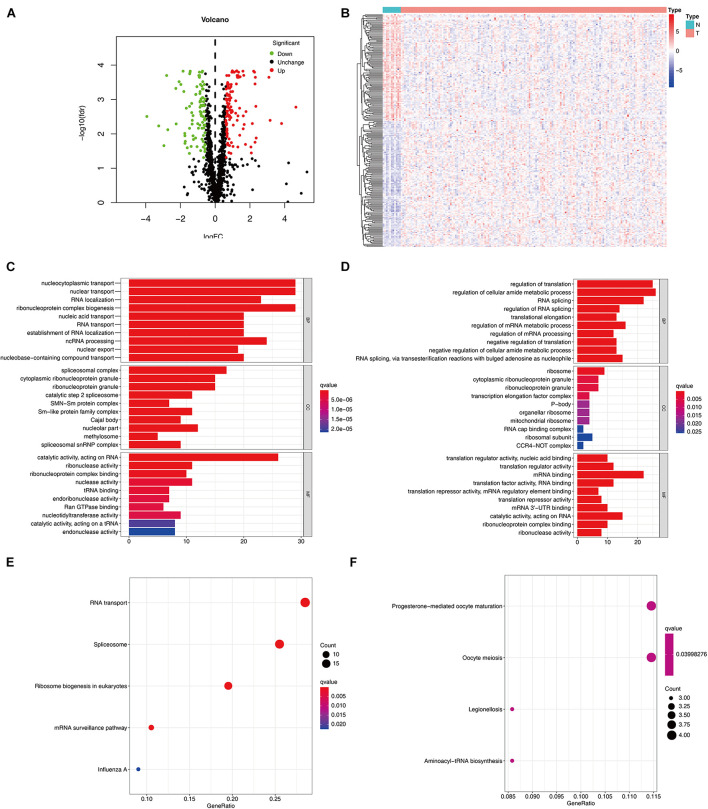
Expression and functional annotation of RBPs in ESCC. Volcano plot **(A)** and heatmap **(B)** of DERBPs in ESCC and normal tissues Results of GO functional annotation analysis of upregulated **(C)** and downregulated **(D)** DERBPs, Results of KEGG pathways enrichment analyses of upregulated **(E)** and downregulated **(F)** DERBPs. The dot size represents the enriched gene number. RBPs, RNA binding proteins GO, Gene ontology; KEGG, Kyoto Encyclopedia of Genes and Genomes.

The detailed workflow of this research is shown in Figure 1. The characteristics of DERBPs from TCGA–ESCC were defined. Multiple indicators were utilized for verification. The mRNA expression data and corresponding clinical information of 162 ESCC tissue samples and 11 non-tumor samples were downloaded from TCGA database (Table 1). After extracting the expression values of 1495 RBPs, the DERBPs were obtained, and the expression patterns of the DERBPs in ESCC and non-tumor tissues were shown by volcano map and thermogram. In ESCC, 218 differentially expressed genes were found, among which 100 were downregulated and 118 were upregulated (Figures 2A,B). Functional enrichment analysis was performed to analyze the biological characteristics of DERBPs. Figures 2C–F and Supplementary Table 1 summarize the GO terms and KEGG pathway enrichment of these genes. Our results show that in the biological processes related to esophageal cancer, upregulated RBPs are significantly enriched nucleocytoplasmic transport, nuclear transport, RNA localization, ribonucleoprotein complex biogenesis, and nucleic acid transport. The downregulated RBPs are significantly enriched in regulation of translation, regulation of cellular amide metabolic process, RNA splicing, regulation of RNA splicing, and translational elongation. In terms of molecular function, upregulated RBPs are significantly enriched in spliceosomal complex, cytoplasmic ribonucleoprotein granule, ribonucleoprotein granule, catalytic step 2 spliceosome, and SMN-Sm protein complex, whereas downregulated RBPs are significantly enriched in ribosome, cytoplasmic ribonucleoprotein granule, ribonucleoprotein granule, transcription elongation factor complex, and P-body. The findings show that upregulated RBPs are mainly enriched in catalytic activity, acting on RNA, ribonuclease activity, ribonucleoprotein complex binding, nuclease activity, and TRNA binding, whereas downregulated RBPs are significantly enriched in translation regulator activity, nuclear acid binding, translation regulator activity, mRNA binding, translation factor activity, RNA binding and translation reporter activity, and mRNA regulatory element binding. In addition, the KEGG pathway enrichment analysis of the DERBPs showed that upregulated RBPs are enriched in RNA transport, spliceosome, ribosome biogenesis in eukaryotes, mRNA surveillance pathway, and Influenza A, whereas downregulated RBPs are enriched in progesterone mediated oocyte formation, legionellosis, oocyte meiosis, and aminoacyl-tRNA biosynthesis.

**TABLE 1:** Clinicopathological parameters of ESCC patients in the TCGA database.

Clinical parameters	Variable	Total (185)	Percentages (%)
**Age**	≤65	113	61.1%
	>65	72	38.9%
**Gender**	Female	27	14.6%
	Male	158	85.4%
**Tumor grade**	G1	19	10.3%
	G2	77	41.6%
	G3	49	26.5%
	GX	40	21.6%
**Pathological stage**	Stage I	21	11.4%
	Stage II	86	46.5%
	Stage III	66	35.7%
	Stage IV	12	6.5%
**Stage T**	T0	2	1.1%
	T1	32	17.3%
	T2	44	23.8%
	T3	102	55.1%
	T4	5	2.7%
**Stage M**	M0	150	81.1%
	M1	12	6.5%
	MX	23	12.4%
**Stage N**	N0	80	43.2%
	N1	74	40.0%
	N2	21	11.4%
	N3	8	4.3%
	NX	2	1.1%
**Survival status**	Dead	77	41.6%
	Alive	108	58.4%

### Construction of PPI Network and Screening of Key Modules

**FIGURE 3:**
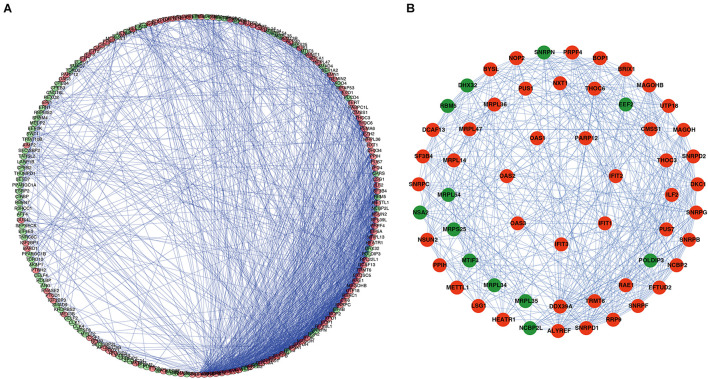
PPI network and module analysis. **(A)** PPI network of DERBPs; **(B)** three critical modules from the PPI network. The outer circle is critical module 1, the middle circle is critical module 2, and the inner circle is critical module 3. Green circles: Downregulation RBPs, Red circles: Upregulation RBPs PPI, Protein–protein interaction RBPs, RBPs, RNA binding proteins.

A PPI network was constructed using STRING database and Cytoscape software to understand the potential molecular functions of these DERBPs in ESCC better. This PPI network contains 197 nodes and 1281 edges (Figure 3A). Subsequently, the coexpression network was further analyzed, the plugin mode in Cytoscape was to detect potential key modules, and the first three important modules were identified (Figure 3B). Module 1 included 31 nodes and 232 edges, Module 2 included 21 nodes and 61 edges, and Module 3 included 7 nodes and 21 edges. Functional enrichment analysis showed that the gene of Module 1 is mainly enriched in spliceosome, mRNA surveillance pathway, and RNA transport. The gene of Module 2 is significantly enriched in mitochondrial gene expression, translational elongation, and mitochondrial translation. However, the gene of Module 3 is significantly enriched in Type I interferon signaling pathway, regulation of protein binding, and response to exogenous dsRNA (Supplementary Table 2).

### Construction and Verification of the ESCC Prognostic Model

**FIGURE 4:**
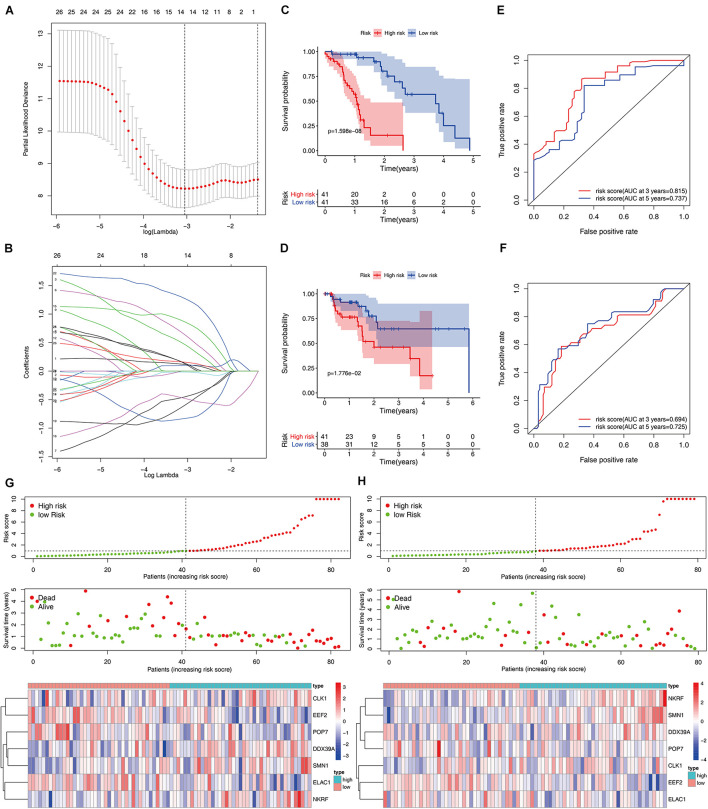
Construction and validation of the ESCC prognostic risk model. **(A,B)** Lasso regression using prognosis-significant RBPs **(C,D)** K–M analysis of the high-risk and low-risk patients in the training group and testing group **(E,F)** 3- and 5-years ROC curves in the training group and testing group. Risk score distribution of patients with ESCC and different risks in the training group (**G**, Top) and testing group (**H**, Top) (low, green; high, red). Dot plots showing the survival time and risk score in training group (**G**, Middle) and testing group (**H**, Middle) Heatmap of the seven key gene expression profiles in the training group (**G**, Bottom) and testing group (**H**, Bottom) (low, blue; high, red).

A total of 197 DERBPs were identified from the PPI network. A prognostic model in the training set of patients with esophageal cancer was established to explore the relationship between these RBPs and prognosis. Initially, univariate Cox regression analysis was performed to obtain genes significantly related to the prognosis, and then Lasso regression and multivariate Cox regression were used to generate a seven-gene model (CLK1, DDX39A, EEF2, ELAC1, NKRF, POP7, and SMN1), as shown in Table 2 and Figures 4A,B. This model was used to calculate the risk score of each patient, and the results showed that CLK1, DDX39A, NKRF, and SMN1 are positive risk-related genes, and EEF2, ELAC1, and POP7 are negative risk-related genes. Then, the patients were divided into high-risk group and low-risk group according to the median risk score, and K–M survival analysis was carried out on the training set and testing set. The results showed that the overall survival time of patients with higher risk score is significantly worse than that of patients with lower risk score in ESCC data set (Figures 4C,D). In addition, the ROC curve was constructed to evaluate the accuracy of the model. The area under the ROC curve of the training group and the testing group is significant (3-years AUC, 0.815 vs. 0.694; 5-years AUC, 0.737 vs. 0.725) (Figures 4E,F). In addition, all patients with ESCC were ranked according to the risk score to analyze the distribution of survival. The survival status of patients with different risk scores can be seen from the scatter plot, and risk score increases the mortality of patients. Thermography showed that the expression of RBPs is associated with the increase of risk score (Figures 4G,H).

**TABLE 2:** The seven selected RNA binding proteins.

Id	Coef	HR	HR.95L	HR.95H	*P*-value
CLK1	0.5651	1.7596	0.8947	3.4608	0.1015
DDX39A	1.3296	3.7797	1.4486	9.8621	0.0066
EEF2	–0.9832	0.3741	0.1489	0.94	0.0365
ELAC1	–1.6889	0.1847	0.054	0.6319	0.0071
NKRF	1.4268	4.1653	1.8453	9.402	0.0006
POP7	–1.2293	0.2925	0.1365	0.6269	0.0016
SMN1	2.1382	8.4843	2.253	31.95	0.0016

### Prognosis Model of Patients With ESCC Is Independently Related to OS

**FIGURE 5:**
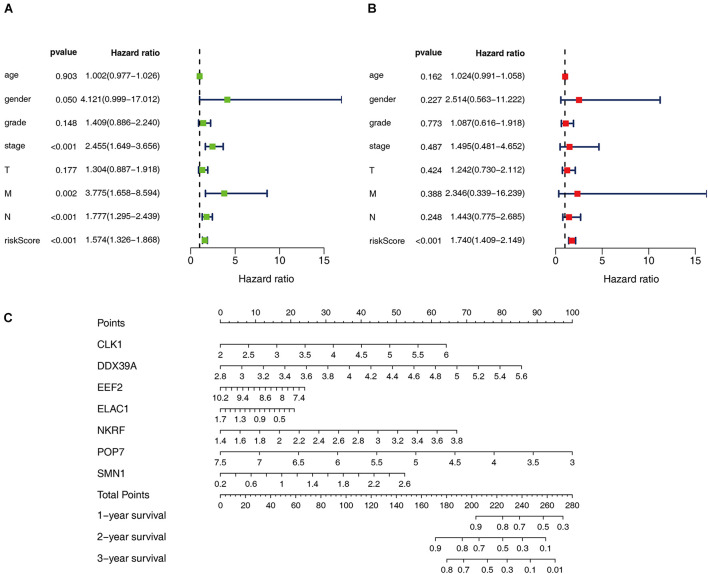
Prognostic value of different clinical parameters. **(A,B)** Univariate and multivariate Cox regression were performed to calculate HR and 95% CI of RBPs. **(C)** Nomogram for predicting 1-, 3-, and 5-years OS of patients with ESCC.

Cox regression analysis was used to analyze the correlation between clinical parameters such as age, gender, histological grade, pathological TMN stage, risk score, and OS. Univariate Cox regression analysis showed that the pathological M, N staging, and risk score of patients with ESCC are related to OS (*P* < 0.05). However, multiple regression analysis found only the risk score is an independent prognostic factor related to OS (*P* < 0.05), as shown in Figure 5. Moreover, seven RBPs were combined to construct a quantitative model for the prognosis of patients with ESCC. Based on the multivariate Cox analysis, the point scale in the nomogram was used to assign points to each variable. A horizontal line was drawn to determine the point of each variable, and the total point of each patient was calculated by summing the points of all variables and then normalizing it to a distribution of 0–100. By drawing a vertical line between the total point axis and each prognostic axis, the estimated survival rate of patients with ESCC at 1, 3, and 5 years can be calculated, which is helpful for relevant practitioners to make clinical decisions for patients with ESCC. Our results indicated that the established specific prognostic model can be used to predict OS in patients with ESCC.

### Comprehensive Analysis of RBP Prognostic Model Genes

**FIGURE 6:**
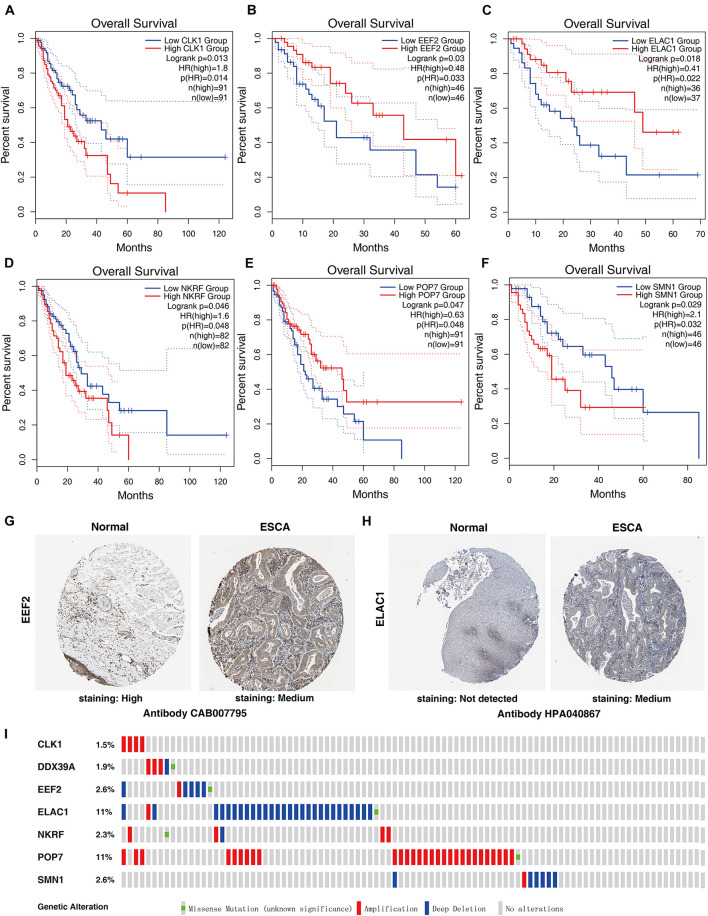
Comprehensive analyses of RBPs in the prognostic model. K–M analyses of **(A)** CLK1, **(B)** EEF2, **(C)** ELAC1, **(D)** NKRF, **(E)** POP7, and **(F)** SMN1 in patients with ESCC. The protein levels of **(G)** EEF2 and **(H)** ELAC1 were determined by immunohistochemistry using indicated antibodies in HPA database, and the staining strengths were annotated as Not detected, Low, Medium, and High. The bar plots indicate the number of samples with different staining strengths in the HPA database. **(I)** Copy number alterations and mRNA expression alterations of seven RBPs in the prognostic model are shown in OncoPrint database.

Seven genes were obtained from the prognostic model, and then the prognostic value of the selected genes was further evaluated in other databases. Correlation analysis of the six selected genes in the timer data showed that most genes are closely related in mRNA expression (Supplementary Figure 1). K–M analysis was performed on the GEPIA2 database. The results showed that in ESCC, CLK1, NKRF, and SMN1 are positively correlated with OS, and EEF2, ELAC1, and POP7 are negatively correlated with OS (Figures 6A–F). In general, the results of K–M analysis are consistent with those of univariate Cox analysis, which means that most genes are implanted into specific prognostic models and have strong predictive ability. Next, the HPA database was used to analyze the protein expression patterns of genes in the prognostic model. The results showed that EEF2 protein is highly expressed in normal esophageal tissues but moderately expressed in esophageal cancer tissues (Figure 6G). The expression of ELAC1 protein was not detected in normal esophageal tissues, but it was moderately expressed in esophageal cancer tissues (Figure 6H). The copy number variation (CNV) and mRNA expression changes of these genes were examined using the cBioPortal database (Figure 6I). The results showed that CNVs are involved in the change of mRNA expression of these genes. POP7 showed the highest changes in CNVs and mRNA expression in the entire analyzed sample, which indicates that CNVs may as a potential driving force for the changes in the mRNA expression of these genes.

### Independent Verification and GSEA

**FIGURE 7:**
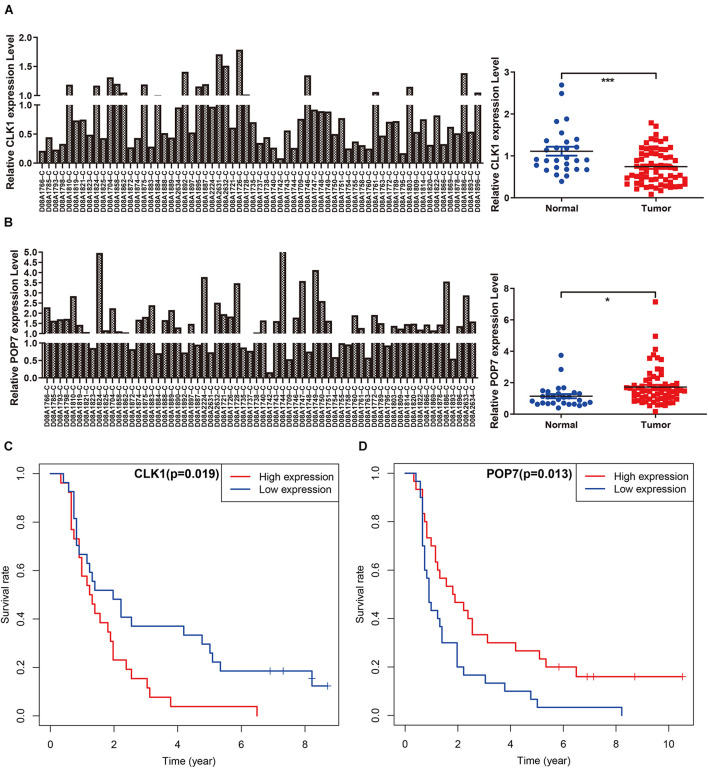
Independent verification in cDNA microarray of ESCC. **(A,B)** Expression of CLK1 and POP7 in esophageal carcinoma and matched adjacent normal tissues. **(C,D)** Survival analysis of CLK1 and POP7 in esophageal carcinoma. *P < 0.05 and ***P < 0.001.

**FIGURE 8:**
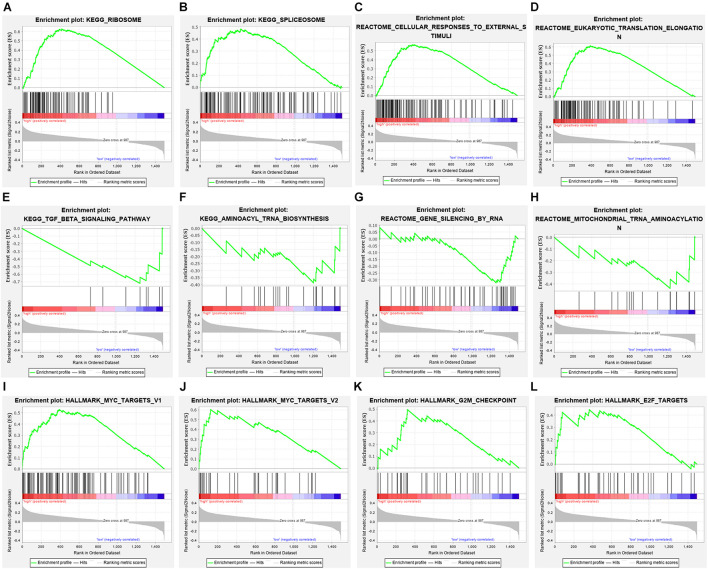
Gene set enrichment analysis in high-risk and low-risk patients with ESCC. **(A)** KEGG_RIBOSOME, **(B)** KEGG_SPLICEOSOME, **(C)** CELLULAR_RESPONSES_TO_EXTERNAL_STIMULI, **(D)** EUKARYOTIC_TRANSLATION_ELONGATION, **(E)** TGF_BETA_SIGNALING_PATHWAY, **(F)** AMINOACYL_TRNA_BIOSYNTHESIS, **(G)** GENE_SILENCING_BY_RNA, **(H)** MITOCHONDRIAL_TRNA_AMINOACYLATION **(I)** HALLMARK_MYC_TARGETS_V1, **(J)** HALLMARK_MYC_TARGETS_V2, **(K)** HALLMARK_G2M_CHECKPOINT, **(L)** HALLMARK_E2F_TARGETS.

Among the seven gene models, studies on CLK1 and POP7 were few. Our focus was on the expression of CLK1 and POP7 in esophageal cancer. First, the expressions of CLK1 and POP7 were compared in 28 pairs of primary esophageal cancer and non-cancerous tissues from the cDNA array. Consistent with our previous results, compared with non-cancerous tissues, CLK1 mRNA levels are lower in esophageal cancer, and POP7 mRNA levels are higher (Figures 7A,B). Survival analysis showed that the higher the expression of CLK1 in esophageal cancer tissues is, the worse the prognosis of patients (Figure 7C); the lower the expression of POP7 is, the worse the prognosis of patients (Figure 7D). These results suggested that CLK1 may play a role in promoting cancer in esophageal cancer, and POP7 may play a role in suppressing cancer in esophageal cancer. We intend to use GSEA to explain the rich characteristics and pathways between high-risk and low-risk patients because our findings revealed that high-risk and low-risk patients have significant prognostic differences in OS (Figure 8). The GSEA enrichment results showed that the high-risk group is enriched in RIBOSOME SPLICEOSOME, CELLULAR_RESPONSES_TO_EXTERNAL_STIMULI, EUKA RYOTIC_TRANSLATION_ELONGATION, HALLMARK_ MYC_ TARGETS_V1, HALLMARK_MYC_TARGETS_V2, HALLMARK_G2M_CHECKPOINT, and HALLMARK_E2F_ TARGETS, whereas the low-risk group is enriched in TGF_BETA_SIGNALING_PATHWAY, AMINOACYL_TRNA_ BIOSYNTHESIS, GENE_SILENCING_BY_RNA, and MITOCHONDRIAL_TRNA_AMINOACYLATION. Several studies showed that these pathways are related to the occurrence and development of esophageal cancer. The GSEA results suggested that RBPs related signals are related to the development and progression of ESCC.

## Discussion

In this study, bioinformatic analysis was used to study the expression of RBPs in the ESCC database deeply, determine the prognostic-related RBPs, and develop a model for the prognosis of ESCC, which may be helpful in the development of biomarkers for diagnosis and prognosis. First, the DERBPs between ESCC and non-tumor tissues were screened, the PPI networks were established, and three key modules were obtained. Considering that these genes may be closely related to the occurrence of ESCC, GO, and KEGG analyses were performed. The module analysis of each network of PPI showed that DERBPs are mainly enriched in spliceosome, mRNA surveillance pathway, and RNA transport mitochondrial gene expression. The spliceosome is mainly composed of small molecules of nuclear RNA and protein. The precursor messenger RNA is formed by multiple introns and exon intervals. Introns must be removed, and exons must be connected by the role of spliceosome before they can be transformed into mature messenger RNA, thus translating into proteins to play a biological effect (JQN et al., 2020; Ochi and Ogawa, 2021). The abnormality of RNA splicing is related to the occurrence and development of tumors, and becomes a target of tumor treatment. RNA plays an important role in organism evolution, genetic information translation, gene expression, and cell function. Research has proven that the abnormal regulation of RNA transport, processing, translation, and catabolic processes is related to the occurrence and development of many diseases (Sciarrillo et al., 2020; Kolathur, 2021; Liu and Rabadan, 2021). In addition to being the place where the cells generate energy, mitochondria also encode 13 polypeptide chains, 22 tRNA molecules, and 2 rRNA molecules that participate in the oxidative respiratory chain of mitochondria (Bogenhagen and Haley, 2020; Lee et al., 2021). The abnormal expression of mitochondrial gene affects the utilization of oxygen, which is closely related to a series of pathophysiological processes. Studies have verified that the abnormal expression of mitochondrial and tumor mitochondrial-encoded gene is closely related to tumors in the liver, breast, colon, and other tissues (Huang et al., 2020; Boran et al., 2021). In most tumors, the number of mitochondria decreases, but the overall expression of mitochondrial oxidative respiratory chain coding gene increases (Penta et al., 2001). The results showed that the expression levels of NADH dehydrogenase, ATP synthetase 6, cytochrome oxidase B, and cytochrome oxidase I and III genes are upregulated in papillary thyroid carcinoma, whereas the expression of nuclear encoded mtTFA is not significantly changed (Haugen et al., 2003). These results indicated that RBPs affect the growth of tumor cells by regulating various biological processes.

Then, a model was established by univariate Cox regression and Lasso regression, and seven OS-related risk genes (CLK1, DDX39A, EEF2, ELAC1, NKRF, POP7, and SMN1) were finally identified. Further, the specific prognosis model (OS model) was constructed, and the model can provide accurate prediction for the prognosis of patients with ESCC. Multivariate Cox regression analysis of the prognostic model and other clinical parameters showed that the model could independently predict the prognosis of patients with ESCC. In addition, GESA enrichment analysis showed that ribosome and MYC related signaling pathways are over activated in high-risk patients. Studies have shown that ribosomal protein is abnormally expressed in a variety of tumors, and affects tumor cell apoptosis, senescence, growth, invasion, and drug and radiotherapy resistance through various mechanisms (Awah et al., 2020; Babaian et al., 2020). The proliferation of tumor cells is positively correlated with protein synthesis. Several tumor suppressors indirectly regulate cell proliferation by interfering with ribosome synthesis. Inactivation of ribosomal protein or p53 gene promotes the synthesis of ribosomal protein and leads to cell proliferation (Ibáñez-Cabellos et al., 2020). Carcinogenic factors facilitate cell proliferation by promoting the synthesis of ribosomal protein. In tumor evolution, tumor suppressor factors and carcinogenic factors jointly regulate the synthesis of ribosomal protein and determine the direction of cell development. The MYC gene family of nuclear oncogenes mainly encode proteins, and their activation and mutation can lead to cell canceration. Current research on MYC shows that its oncoprotein is abnormally expressed in oral and esophageal tumors, and its amplification is closely related to the occurrence and development of esophageal tumors (Sproll et al., 2018; McEvoy et al., 2021).

The enrichment analysis of the low-risk group suggests that it is enriched in classic tumor signaling pathways such as transforming growth factor-β (TGF-β) signaling pathway. In mammals, the TGF-β signaling pathway can affect cell differentiation, proliferation, migration, extracellular matrix remodeling, apoptosis, and other biological processes (Wharton and Derynck, 2009; Yan et al., 2014; Jia and Meng, 2021). In general, the binding of TGF-β family ligands to the extracellular domain of TGF-β receptor triggers the activation of downstream effectors of classical Smads Protein signal, leading to the transcription of important genes related to tissue homeostasis, tumor growth, and progression. Unexpectedly, TGF-β signaling seems to have a dual role in regulating tumorigenesis: it is a growth inhibitor in the early stage but promotes tumor progression and metastasis in the late stage (Morikawa et al., 2016; Zhao et al., 2018). This dual effect has been confirmed in ESCC and EAC. In the early stage of esophageal cancer, the TGF-β signaling pathway seems to have an inhibitory effect on tumor growth. EAC and ESCC cell lines reduce the TGF-β responsiveness by downregulating the expression of Smad4 or c-MYC (Batlle and Massagué, 2019). Accordingly, Smad4 expression gradually decreases in EAC metaplasia hyperplasia adenocarcinoma. After the recovery of Smad4 expression, the inhibition of proliferation recovers (Onwuegbusi et al., 2006). Unexpectedly, the results of TGF-β in ESCC are inconsistent. DACH1 methylation leads to downregulation of TGF-β or decreased expression of Smad4, which is related to increased depth of invasion, late tumor stage, and poor differentiation. The downregulation of TGF-β caused by proteasome degradation inhibits tumor growth and invasion *in vivo*. Nevertheless, the results of studies on patients with ESCC still support the tumor suppressor effect of TGF-β, and reduced signal transduction is associated with more aggressive tumor characteristics and worse prognosis (Chen et al., 2018).

The literature search of the seven selected model genes found few reports on the pathogenesis of CLK1, POP7, and ESCC. Therefore, they are expected to be new biological targets for ESCC treatment. CLK1 is a mitochondrial hydroxylase, an important regulatory component that encodes mitochondrial enzymes, which are involved in the biosynthesis of coenzyme Q, which is a necessary coaction factor in many redox reactions including mitochondrial respiratory chain (Takahashi et al., 2012). CLK1 affects many physiological processes; the cytoplasmic reactive oxygen species (ROS) level of cells with CLK1 gene mutations is significantly reduced, which leads to reduced lipoprotein oxidative damage and decreased activation of intracellular carcinogenic signals (Lapointe and Hekimi, 2008; Gu et al., 2017). Previous studies reported that CLK1 gene mutation in cryptodendromyonema is responsible for slowing down the growth of the nematodes and prolonging their life (Felkai et al., 1999). Studies have found that downregulating the CLK1 gene in glioma GL261 cells can inhibit mitochondrial function, reduce AMPK phosphorylation levels, activate the mTOR signaling pathway, promote the upregulation of HIF-1α in tumor cells, and enhance the sensitivity of GL261 cells to chemotherapy drugs. At present, no research focuses on the relationship between CLK1 and esophageal cancer (Zhang et al., 2017). Our results revealed that the level of CLK1 mRNA in esophageal cancer is relatively low. Survival analysis showed that the higher expression of CLK1 in esophageal cancer tissue is, the worse prognosis of patients, suggesting that CLK1 may play a role in promoting esophageal cancer. Therefore, indepth analysis of the changes and mechanisms of CLK1 is expected to provide new molecular markers for the early detection of ESCC. POP7 is a protein coding gene. Studies have found that POP7-related diseases include retinitis pigmentosa and hairy tongue. Among its related pathways are tRNA processing and gene expression (Perederina et al., 2007). No research studied the relationship between POP7 and esophageal cancer. Our results show that the level of POP7 mRNA in esophageal cancer is relatively higher, and the lower expression of CLK1 is, the worse prognosis of patients in esophageal cancer, suggesting that POP7 may play a role in promoting progression of esophageal cancer. Therefore, analysis of POP7 in the development of ESCC is expected to provide a new molecular marker for early detection.

RBP has a diverse structure of RNA binding domains and participates in the regulation of multiple transcriptional mechanisms. Dysfunction of RBPs in cancers cause abnormal translation and overexpression of mRNA, and promote tumor growth, invasion and angiogenesis. Specific prognostic models of RBPs based on the comprehensive analysis of RBP expression profile and corresponding clinical characteristics provide new targets for the treatment and intervention of esophageal cancer. Therefore, further research should focus on clarifying the accuracy of the comprehensive analysis of model gene expression and the mechanism by which RBPs regulate the occurrence and development of ESCC.

## Data Availability Statement

The datasets presented in this study can be found in online repositories. The names of the repository/repositories and accession number(s) can be found in the article/Supplementary Material.

## Author Contributions

XPY, BAH, and HYS: conception and design. MLY and XC: development of methodology. ZHH, YZ, KL, and HGS: analysis and interpretation of data. XPY, MLY, and DH: writing – review and revision of the manuscript. XC: study supervision. All authors contributed to the article and approved the submitted version.

## Conflict of Interest

The authors declare that the research was conducted in the absence of any commercial or financial relationships that could be construed as a potential conflict of interest.

## Publisher’s Note

All claims expressed in this article are solely those of the authors and do not necessarily represent those of their affiliated organizations, or those of the publisher, the editors and the reviewers. Any product that may be evaluated in this article, or claim that may be made by its manufacturer, is not guaranteed or endorsed by the publisher.

## Footnotes

1
http://www.STRING-db.org/

2
http://gepia2.cancer-pku.cn/

3
https://www.proteinatlas.org/

4
http://www.cbioportal.org/

## Supplementary Material

Supplementary Figure 1 Relationship between the expression of RBPs in the prognostic model. Cor: Correlation coefficient. The value range of correlation coefficient is (–1, 0) or (0, 1). A value range of (–1, 0) means negative correlation, and a value range of (0, 1) means positive correlation.

Supplementary Table 1 GO and KEGG analysis of the differentially expressed RBPs.

Supplementary Table 2 The results of GO and KEGG analysis in 3 modules.

Supplementary Table 3 Clinicopathological parameters of all patients with ESCC.

## Abbreviations

ESCC: Esophageal squamous cell carcinoma
RBPs: RNA binding proteins
DERBPs: Differential-expressed RNA binding proteins
OS: Overall survival
TIMER: Tumor immune estimation resource
GEPIA: Gene expression profiling interactive analysis
LASSO: Least absolute shrinkage and selection operator
CNV: Copy number variation
GSEA: Gene set enrichment analysis.

## References

[B1] Arnold M. Pandeya N. Byrnes G. Pag R. Stevens G. A. Ezzati P. M. (2015). Global burden of cancer attributable to high body-mass index in 2012: a population-based study. *Lancet Oncol.* 16 36–46. 10.1016/S1470-2045(14)71123-4 25467404 PMC4314462

[B2] Attig J. Ule J. (2019). Genomic accumulation of retrotransposons was facilitated by repressive RNA-binding proteins: a hypothesis. *Bioessays* 41:e1800132. 10.1002/bies.201800132 30706962

[B3] Awah C. U. Chen L. Bansal M. Mahajan A. Winter J. Lad M. (2020). Ribosomal protein S11 influences glioma response to TOP2 poisons. *Oncogene* 39 5068–5081. 10.1038/s41388-020-1342-0 32528131 PMC7646677

[B4] Babaian A. Rothe K. Girodat D. Minia I. Djondovic S. Milek M. (2020). Loss of m1acp3Ψ ribosomal RNA modification is a major feature of cancer. *Cell Rep.* 31:107611. 10.1016/j.celrep.2020.107611 32375039

[B5] Batlle E. Massagué J. (2019). Transforming growth factor-β signaling in immunity and cancer. *Immunity* 50 924–940. 10.1016/j.immuni.2019.03.024 30995507 PMC7507121

[B6] Bogenhagen D. F. Haley J. D. (2020). Pulse-chase SILAC-based analyses reveal selective oversynthesis and rapid turnover of mitochondrial protein components of respiratory complexes. *J. Biol. Chem.* 295 2544–2554. 10.1074/jbc.RA119.011791 31974161 PMC7049976

[B7] Boran T. Akyildiz A. G. Jannuzzi A. T. Alpertunga B. (2021). Extended regorafenib treatment can be linked with mitochondrial damage leading to cardiotoxicity. *Toxicol. Lett.* 336 39–49. 10.1016/j.toxlet.2020.11.003 33166663

[B8] Chen X. Wang L. Li P. Song M. Qin G. Gao Q. (2018). Dual TGF-β and PD-1 blockade synergistically enhances MAGE-A3-specific CD8+ T cell response in esophageal squamous cell carcinoma. *Int. J. Cancer* 143 2561–2574. 10.1002/ijc.31730 29981155

[B9] Dang H. Takai A. Forgues M. Pomyen Y. Mou H. Xue W. (2017). Oncogenic activation of the RNA binding protein NELFE and MYC signaling in hepatocellular carcinoma. *Cancer Cell* 32 101.e–114.e. 10.1016/j.ccell.2017.06.002 28697339 PMC5539779

[B10] Felkai S. Ewbank J. J. Lemieux J. Labbé J. C. Brown G. G. Hekimi S. (1999). CLK-1 controls respiration, behavior and aging in the nematode *Caenorhabditis* elegans. *EMBO J.* 18 1783–1792. 10.1093/emboj/18.7.1783 10202142 PMC1171264

[B11] Ferlay J. Colombet M. Soerjomataram I. Mathers C. Parkin D. M. Piñeros M. (2019). Estimating the global cancer incidence and mortality in 2018: GLOBOCAN sources and methods. *Int. J. Cancer* 144 1941–1953. 10.1002/ijc.31937 30350310

[B12] Gerstberger S. Hafner M. Tuschl T. (2014). A census of human RNA-binding proteins. *Nat. Rev. Genet.* 15 829–845. 10.1038/nrg3813 25365966 PMC11148870

[B13] Gu R. Zhang F. Chen G. Han C. Liu J. Ren Z. (2017). Clk1 deficiency promotes neuroinflammation and subsequent dopaminergic cell death through regulation of microglial metabolic reprogramming. *Brain Behav. Immun.* 60 206–219. 10.1016/j.bbi.2016.10.018 27769915

[B14] Hamilton K. E. Chatterji P. Lundsmith E. T. Andres S. F. Giroux V. Hicks P. D. (2015). Loss of stromal IMP1 promotes a tumorigenic microenvironment in the colon. *Mol. Cancer Res.* 13 1478–1486. 10.1158/1541-7786.MCR-15-0224 26194191 PMC4644674

[B15] Harada K. Rogers J. E. Iwatsuki M. Yamashita K. Baba H. Ajani J. A. (2020). Recent advances in treating oesophageal cancer. *F1000Res.* 9:F1000 Faculty Rev-1189. 10.12688/f1000research.22926.1 33042518 PMC7531047

[B16] Hattori A. Buac K. Ito T. (2016). Regulation of stem cell self-renewal and oncogenesis by RNA-binding proteins. *Adv. Exp. Med. Biol.* 907 153–188. 10.1007/978-3-319-29073-7_7 27256386

[B17] Haugen D. R. Fluge Ø Reigstad L. J. Varhaug J. E. Lillehaug J. R. (2003). Increased expression of genes encoding mitochondrial proteins in papillary thyroid carcinomas. *Thyroid* 13 613–620. 10.1089/105072503322239943 12964965

[B18] Huang G. Li H. Zhang H. (2020). Abnormal expression of mitochondrial ribosomal proteins and their encoding genes with cell apoptosis and diseases. *Int. J. Mol. Sci.* 21:8879. 10.3390/ijms21228879 33238645 PMC7700125

[B19] Ibáñez-Cabellos J. S. Seco-Cervera M. Picher-Latorre C. Pérez-Machado G. García-Giménez J. L. Pallardó F. V. (2020). Acute depletion of telomerase components DKC1 and NOP10 induces oxidative stress and disrupts ribosomal biogenesis via NPM1 and activation of the P53 pathway. *Biochim. Biophys. Acta. Mol. Cell Res.* 1867:118845. 10.1016/j.bbamcr.2020.118845 32910990

[B20] Jia S. Meng A. (2021). TGFβ family signaling and development. *Development* 148:dev188490. 10.1242/dev.188490 33712443

[B21] JQN N. Drabarek W. Yavuzyigitoglu S. Medico S. E. Verdijk R. M. Naus N. C. (2020). Spliceosome mutations in uveal melanoma. *Int. J. Mol. Sci.* 21:9546. 10.3390/ijms21249546 33333932 PMC7765440

[B22] Kolathur K. K. (2021). Role of promoters in regulating alternative splicing. *Gene* 782:145523. 10.1016/j.gene.2021.145523 33667606

[B23] Lapointe J. Hekimi S. (2008). Early mitochondrial dysfunction in long-lived Mclk1+/- mice. *J. Biol. Chem.* 283 26217–26227. 10.1074/jbc.M803287200 18635541 PMC3258865

[B24] Lee M. Matsunaga N. Akabane S. Yasuda I. Ueda T. Takeuchi-Tomita N. (2021). Reconstitution of mammalian mitochondrial translation system capable of correct initiation and long polypeptide synthesis from leaderless mRNA. *Nucleic Acids Res.* 49 371–382. 10.1093/nar/gkaa1165 33300043 PMC7797035

[B25] Lin H. N. Chen L. Q. Shang Q. X. Yuan Y. Yang Y. S. (2020). A meta-analysis on surgery with or without postoperative radiotherapy to treat squamous cell esophageal carcinoma. *Int. J. Surg.* 80 184–191. 10.1016/j.ijsu.2020.06.046 32659390

[B26] Liu Z. Rabadan R. (2021). Computing the role of alternative splicing in cancer. *Trends Cancer* 7 347–358. 10.1016/j.trecan.2020.12.015 33500226 PMC7969404

[B27] Macedo-Silva C. Miranda-Gonçalves V. Henrique R. Jerónimo C. Bravo I. (2019). The critical role of hypoxic microenvironment and epigenetic deregulation in esophageal cancer radioresistance. *Genes (Basel)* 10:927. 10.3390/genes10110927 31739546 PMC6896142

[B28] Mariette C. Finzi L. Piessen G. Van Seuningen I. Triboulet J. P. (2005). Esophageal carcinoma: prognostic differences between squamous cell carcinoma and adenocarcinoma. *World J. Surg.* 29 39–45. 10.1007/s00268-004-7542-x 15599738

[B29] McEvoy C. R. Holliday H. Thio N. Mitchell C. Choong D. Y. Yellapu B. (2021). A MXI1-NUTM1 fusion protein with MYC-like activity suggests a novel oncogenic mechanism in a subset of NUTM1-rearranged tumors. *Lab. Invest.* 101 26–37. 10.1038/s41374-020-00484-3 32873880

[B30] Mikuni H. Yamamoto S. Kato K. (2021). Nivolumab for the treatment of esophageal cancer. *Expert Opin. Biol. Ther.* 21 697–703. 10.1080/14712598.2021.1904887 33736560

[B31] Mongroo P. S. Noubissi F. K. Cuatrecasas M. Kalabis J. King C. E. Johnstone C. N. (2011). IMP-1 displays cross-talk with K-Ras and modulates colon cancer cell survival through the novel proapoptotic protein CYFIP2. *Cancer Res.* 71 2172–2182. 10.1158/0008-5472.CAN-10-3295 21252116 PMC3059369

[B32] Morikawa M. Derynck R. Miyazono K. (2016). TGF-β and the TGF-β Family: context-dependent roles in cell and tissue physiology. *Cold Spring Harb. Perspect. Biol.* 8:a021873. 10.1101/cshperspect.a021873 27141051 PMC4852809

[B33] Ochi Y. Ogawa S. (2021). Chromatin-spliceosome mutations in acute myeloid leukemia. *Cancers (Basel)* 13:1232. 10.3390/cancers13061232 33799787 PMC7999050

[B34] Onwuegbusi B. A. Aitchison A. Chin S. F. Kranjac T. Mills I. Huang Y. (2006). Impaired transforming growth factor beta signalling in Barrett’s carcinogenesis due to frequent SMAD4 inactivation. *Gut* 55 764–774. 10.1136/gut.2005.076430 16368780 PMC1856235

[B35] Ostareck D. H. Ostareck-Lederer A. (2019). RNA-binding proteins in the control of LPS-induced macrophage response. *Front. Genet.* 10:31. 10.3389/fgene.2019.00031 30778370 PMC6369361

[B36] Pelava A. Schneider C. Watkins N. J. (2016). The importance of ribosome production, and the 5S RNP-MDM2 pathway, in health and disease. *Biochem. Soc. Trans.* 44 1086–1090. 10.1042/BST20160106 27528756 PMC4984446

[B37] Penta J. S. Johnson F. M. Wachsman J. T. Copeland W. C. (2001). Mitochondrial DNA in human malignancy. *Mutat. Res.* 488 119–133. 10.1016/s1383-5742(01)00053-9 11344040

[B38] Perederina A. Esakova O. Koc H. Schmitt M. E. Krasilnikov A. S. (2007). Specific binding of a Pop6/Pop7 heterodimer to the P3 stem of the yeast RNase MRP and RNase P RNAs. *RNA* 13 1648–1655. 10.1261/rna.654407 17717080 PMC1986809

[B39] Sciarrillo R. Wojtuszkiewicz A. Assaraf Y. G. Jansen G. Gjl K. Giovannetti E. (2020). The role of alternative splicing in cancer: from oncogenesis to drug resistance. *Drug Resist. Updat.* 53:100728. 10.1016/j.drup.2020.100728 33070093

[B40] Seo D. J. Choi C. (2021). Antiviral bioactive compounds of mushrooms and their antiviral mechanisms: a review. *Viruses* 13:350. 10.3390/v13020350 33672228 PMC7926341

[B41] Sephton C. F. Yu G. (2015). The function of RNA-binding proteins at the synapse: implications for neurodegeneration. *Cell. Mol. Life Sci.* 72 3621–3635. 10.1007/s00018-015-1943-x 26047658 PMC4565867

[B42] Sproll C. Fluegen G. Stoecklein N. H. (2018). Minimal residual disease in head and neck cancer and esophageal cancer. *Adv. Exp. Med. Biol.* 1100 55–82. 10.1007/978-3-319-97746-1_4 30411260

[B43] Takahashi M. Ogawara M. Shimizu T. Shirasawa T. (2012). Restoration of the behavioral rates and lifespan in clk-1 mutant nematodes in response to exogenous coenzyme Q(10). *Exp. Gerontol.* 47 276–279. 10.1016/j.exger.2011.12.012 22244837

[B44] Thrift A. P. (2021). Global burden and epidemiology of Barrett oesophagus and oesophageal cancer. *Nat. Rev. Gastroenterol. Hepatol.* 18 432–443. 10.1038/s41575-021-00419-3 33603224

[B45] Uchida Y. Chiba T. Kurimoto R. Asahara H. (2019). Post-transcriptional regulation of inflammation by RNA-binding proteins via cis-elements of mRNAs. *J. Biochem.* 166 375–382. 10.1093/jb/mvz067 31511872 PMC6804641

[B46] Wharton K. Derynck R. (2009). TGFbeta family signaling: novel insights in development and disease. *Development* 136 3691–3697. 10.1242/dev.040584 19855012

[B47] Yan W. Li J. Chai R. Guo W. Xu L. Han Y. (2014). Combining use of captopril and losartan attenuates the progress of Streptococcus pneumoniae-induced tympanosclerosis through the suppression of TGF-β1 expression. *PLoS One* 9:e111620. 10.1371/journal.pone.0111620 25360706 PMC4216096

[B48] Zeng B. Huang P. Du P. Sun X. Huang X. Fang X. (2021). Comprehensive study of germline mutations and double-hit events in esophageal squamous cell cancer. *Front. Oncol.* 11:637431. 10.3389/fonc.2021.637431 33889545 PMC8056176

[B49] Zhang L. Yang H. Zhang W. Liang Z. Huang Q. Xu G. (2017). Clk1-regulated aerobic glycolysis is involved in glioma chemoresistance. *J. Neurochem.* 142 574–588. 10.1111/jnc.14096 28581641

[B50] Zhao M. Mishra L. Deng C. X. (2018). The role of TGF-β/SMAD4 signaling in cancer. *Int. J. Biol. Sci.* 14 111–123. 10.7150/ijbs.23230 29483830 PMC5821033

