# Supplementary figures and images for: RNA-Binding Proteins CLK1 and POP7 as Biomarkers for Diagnosis and Prognosis of Esophageal Squamous Cell Carcinoma
Source: Front Cell Dev Biol. 2021 Sep 9;9:715027. doi: 10.3389/fcell.2021.715027 (PMC8458940; doi:10.3389/fcell.2021.715027)

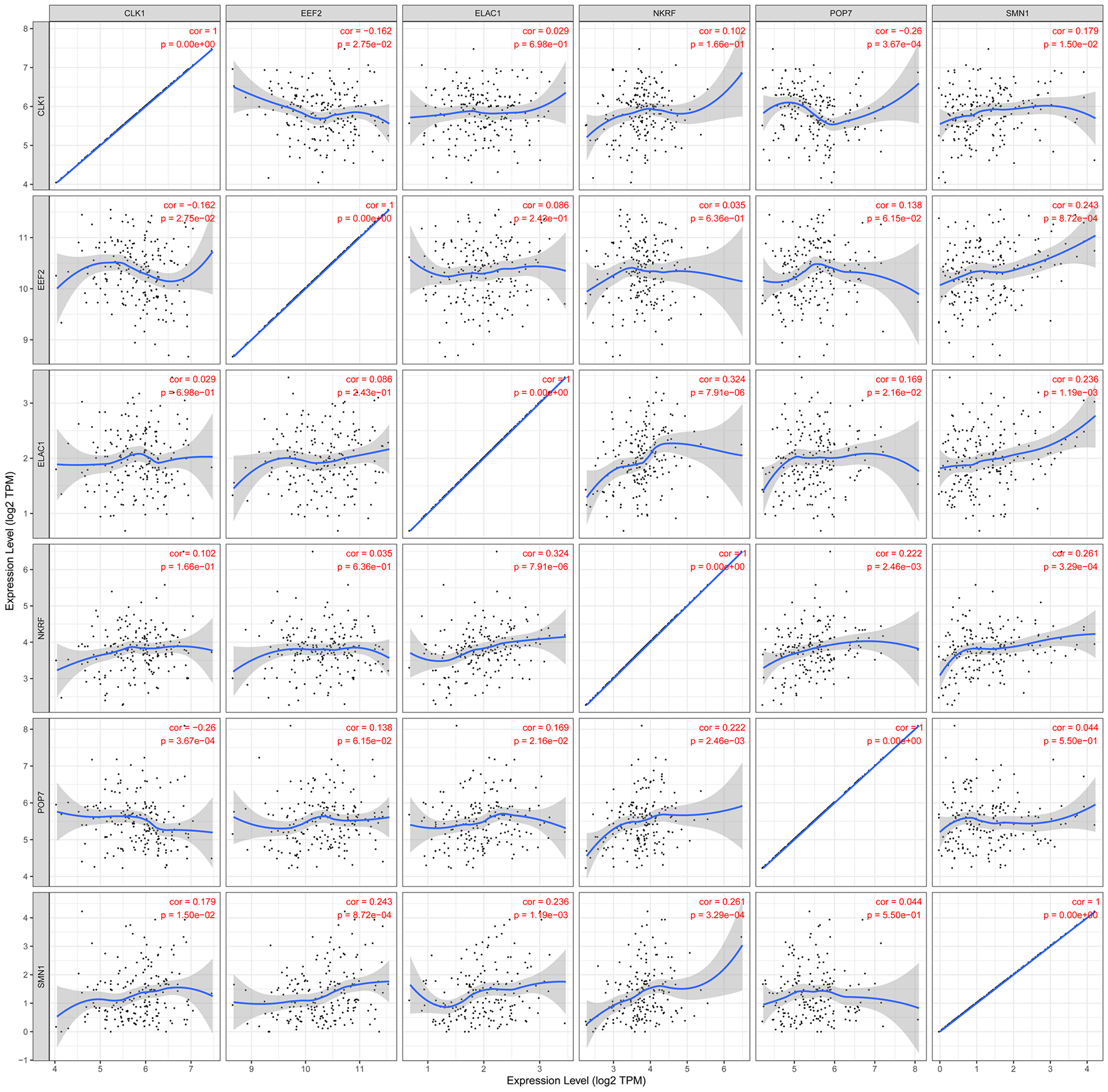

Supplement: Supplementary Figure 1 — Relationship between the expression of RBPs in the prognostic model. Cor: Correlation coefficient. The value range of correlation coefficient is (–1, 0) or (0, 1). A value range of (–1, 0) means negative correlation, and a value range of (0, 1) means positive correlation. [file Image_1.TIF]
